# Comparative Evaluation of Urinary Biomarkers in Wilms Tumor Survivors and Children with Chronic Kidney Disease

**DOI:** 10.3390/ijms26136238

**Published:** 2025-06-27

**Authors:** Pawel Dubiela, Katarzyna Taranta-Janusz, Katarzyna Konończuk, Karolina Konstantynowicz-Nowicka, Adrian Chabowski, Paulina Szymanska-Rozek, Eryk Latoch

**Affiliations:** 1 Department of Regenerative Medicine and Immune Regulation, Medical University of Bialystok, 15-089 Bialystok, Poland; paweldubiela89@gmail.com; 2 Department of Pediatrics and Nephrology, Medical University of Bialystok, 15-089 Bialystok, Poland; katarzyna.taranta@udsk.pl; 3 Department of Pediatrics, Oncology and Hematology, Medical University of Bialystok, 15-089 Bialystok, Poland; kononczukk@gmail.com; 4 Department of Physiology, Medical University of Bialystok, 15-089 Bialystok, Poland; karolina.konstantynowicz-nowicka@umb.edu.pl (K.K.-N.); adrian.chabowski@umb.edu.pl (A.C.); 5 Faculty of Mathematics, Informatics and Mechanics, University of Warsaw, 00-927 Warsaw, Poland; p.szymanska@gmail.com

**Keywords:** biomarkers, CKD, pediatric CKD, Wilms tumor

## Abstract

Wilms tumor (WT), the most common pediatric renal malignancy, shares some clinical and pathological features with chronic kidney disease (CKD). Understanding biomarkers of kidney injury among CKD and WT patients is of high interest due to its potential implications for diagnosis, prognosis, and treatment strategies. This study enrolled twenty pediatric patients with WT (stage I–IV), forty with CKD (stage I–V), and twenty healthy volunteers. Urine samples were collected and six urine biomarkers (calbindin, clusterin, GST-π, IL-18, KIM-1, MCP-1) associated with kidney injury were assessed using the Bio-Plex Pro RBM Human Kidney Toxicity Assays kit (Bio-Plex Manager software 4.0). A comparative analysis of biomarker levels across the three groups revealed distinct patterns. Creatinine levels were notably elevated in CKD (1.32 ± 1.9) compared to WT (0.64 ± 0.26) and the control group. Tested biomarkers were calculated per milligram of urine creatinine, and all the differences among the groups were statistically significant. Pearson’s correlation coefficients showed strong interplay among CKD biomarkers. This study identified variations in biomarker patterns among WT and CKD patients. Understanding biomarker interactions may provide future diagnostic approaches for pediatric kidney conditions.

## 1. Introduction

Wilms tumor (WT), the most prevalent pediatric renal malignancy, and chronic kidney disease (CKD) in children represent critical challenges in pediatric oncology and nephrology, respectively [1,2]. Despite their distinct etiologies and clinical presentations, both conditions necessitate accurate diagnostic tools and prognostic indicators for optimal management. Although CKD in childhood often arises due to a variety of etiological factors, including congenital anomalies of the kidney and urinary tract, genetic conditions, autoimmune disorders, and infectious diseases [3,4,5], WT represents a significant trigger leading to progressive renal damage among childhood cancer survivors during childhood, thereby exacerbating the risk and complexity of CKD onset and progression [2,6]. However, an intriguing question remains: do different underlying pathologies ultimately result in similar patterns of kidney injury in CKD [4,7]?

Biomarkers play an important role in elucidating the pathophysiology of CKD by providing measurable indicators of kidney injury and dysfunction. There are some promising molecules potentially linking CKD and WT. Calbindin plays a multifaceted role in the pathophysiology of CKD, contributing to renal calcium handling, tubular cell survival, and inflammatory responses. Due its physiological role in cellular signaling, it has also been implicated in various cancers and renal pathologies [7]. Similarly, clusterin, a multifunctional glycoprotein, has garnered attention for its roles in apoptosis regulation and drug resistance, prompting exploration of its relevance to both WT and CKD [8,9]. Another interesting molecule is glutathione S-transferase π (GST-π), an enzyme crucial for detoxification and oxidative stress defense. It has been studied in the context of renal carcinogenesis and kidney injury, suggesting potential implications for WT and CKD [10,11]. Another mechanism of renal impairment is related to interleukin-18 (IL-18), a pro-inflammatory cytokine, and kidney injury molecule-1 (KIM-1), a marker of renal injury. Both offer insights into inflammatory pathways and tissue damage mechanisms shared between WT and CKD [12,13]. Monocyte chemoattractant protein-1 (MCP-1), a chemokine involved in immune cell recruitment and inflammation, may provide valuable information about the tumor microenvironment and renal injury processes in both conditions [14,15].

Our investigation aims to elucidate the shared molecular biomarkers profile among both WT survivors and children with CKD due to other nephrological causes. We hypothesize that selected biomarkers, including calbindin, clusterin, glutathione S-transferase π (GST-π), interleukin-18 (IL-18), kidney injury molecule-1 (KIM-1), and monocyte chemoattractant protein-1 (MCP-1), will exhibit alterations indicative of renal injury and inflammation in both conditions.

## 2. Results

### 2.1. Biomarkers Patterns

The study included three groups: WT, CKD, and control. The WT group comprised 20 cancers survivors with a median age of 13 years, ranging from 4 to 18 years. This group had a predominance of females, with 13 females and 7 males. At the time of diagnosis, seven patients (35%) were classified as stage I (WT I), four patients (20%) as stage II (WT II), five patients (25%) as stage III (WT III), and four patients (20%) as stage IV (WT IV). The CKD group consisted of 40 participants, with a median age of 9 years and an age range of 1 to 16 years. This group had a slightly higher number of females (22) compared to males (18). At diagnosis, 14 patients (35%) were classified as stage I, 6 patients (15%) as stage II, 4 patients (10%) as stage III, 4 patients (10%) as stage IV, and 12 patients (30%) as stage V. The control group included 20 participants with an equal distribution of females and males (10 each). Patient characteristics are presented in Table 1.

A comparative analysis of biomarker levels across the three groups was performed. Serum creatinine levels were notably elevated in the CKD group compared to the WT group (1.32 ± 1.9 mg/dL vs. 0.64 ± 0.26 mg/dL). Clusterin levels were substantially higher in the CKD group compared to both the WT and control groups (349 ± 1310 pg/mL vs. 40.8 ± 70 pg/mL in WT, 18.1 ± 21 pg/mL in the control group). Similarly, GST-π levels were markedly elevated in the CKD group compared to the other groups (5.55 ± 20.6 pg/mL vs. 0.847 ± 1.3 pg/mL in WT, 5.24 ± 10 pg/mL in the control group). KIM-1 levels also showed significant differences, with the CKD group exhibiting higher levels compared to the WT and control groups (0.378 ± 1.61 pg/mL vs. 0.244 ± 0.314 pg/mL in WT, 0.173 ± 0.168 pg/mL in the control group). Conversely, calbindin levels were lower in the CKD group compared to the WT and control groups (39.6 ± 81.8 pg/mL vs. 59.8 ± 57.1 pg/mL in WT, 75.2 ± 87.4 pg/mL in the control group). IL-18 and MCP-1 levels exhibited variations across the groups, indicating potential differences in inflammatory and chemotactic responses. Results are presented in Table 2.

Despite the apparent differences in biomarker patterns across the groups, ANOVA analysis revealed that these differences were not statistically significant. The initially observed elevation of clusterin in CKD patients was potentially an artifact due to outliers, with two individuals in the CKD group exhibiting results over 6000 pg/mL, significantly biasing the overall group mean. Upon removal of these outliers, the mean reduced to 80 pg/mL, which was not statistically different from the means of 40.8 pg/mL and 18.1 pg/mL observed in the other groups. A similar effect was observed with GST-π results, where discrepancies were attributed to outliers within the CKD and control groups, strongly biasing the group means. IL-18 was not considered due to the limited data available for the control group. Furthermore, a simple *t*-test comparing the WT and CKD groups did not reveal statistically significant differences. All ANOVA results are detailed in Table 2.

### 2.2. Pattern Assessment of Normalized Urine Biomarkers Using Creatinine Calibration

Biomarker levels, normalized per milligram of urine creatinine in the WT and CKD patients, revealed significant patterns among the groups. Clusterin was higher in the CKD group (0.475 [0.109–6.607]) than in WT (0.114 [0.013–0.872]) with a *p*-value of <0.001. Similarly, GST-π was elevated in CKD (0.004 [0.002–0.015]) compared to WT (0.007 [0.002–0.013]) with a *p*-value of <0.001.

KIM-1 levels also differed, being higher in CKD (0.003 [0.001–0.004]) compared to WT (0.001 [0.0005–0.0047]) with a *p*-value of <0.001. Conversely, Calbindin was lower in CKD (0.348 [0.086–0.601]) compared to WT (0.237 [0.129–0.808]) with a *p*-value of <0.001.

IL-18 and MCP-1 showed variations; IL-18 in CKD was 0.0002 [0.0001–0.0004] compared to WT’s 0.0001 [0.00009–0.0003] with a *p*-value of 0.009. MCP-1 was higher in CKD (0.003 [0.001–0.013]) than WT (0.0009 [0.0007–0.002]) with a *p*-value of <0.001. Results are presented in Table 3.

### 2.3. Correlation Between Tested Biomarkers

The interplay of tested kidney-damage biomarkers was explored using Pearson’s product–moment correlation coefficients. Strong positive correlations were observed among the parameters in the CKD group. The WT group exhibited fewer and less pronounced correlations. In the control group, only two correlations were identified, with the GST-π and KIM-1 correlation being particularly robust. Notably, no correlation was found between creatinine and any other parameter in any of the three groups.

**Figure 1:**
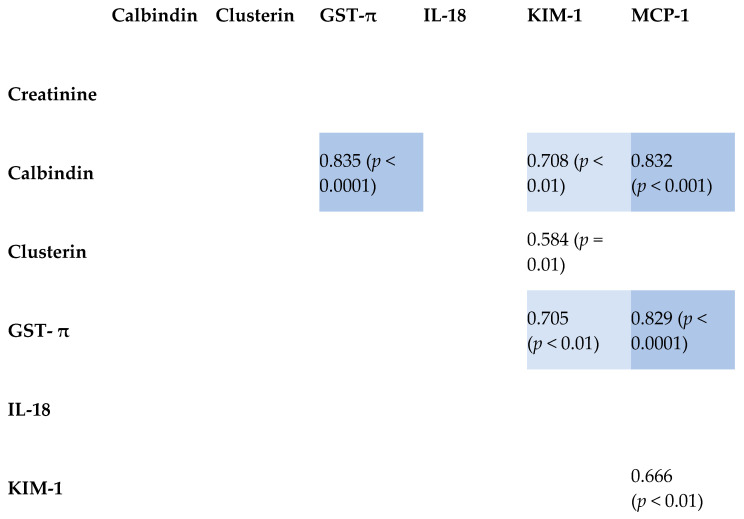
Correlation matrix heatmap. Pearson’s product–moment correlation coefficients along with raw *p*-values calculated for the six parameters in the group of patients diagnosed with WT.

**Figure 2:**
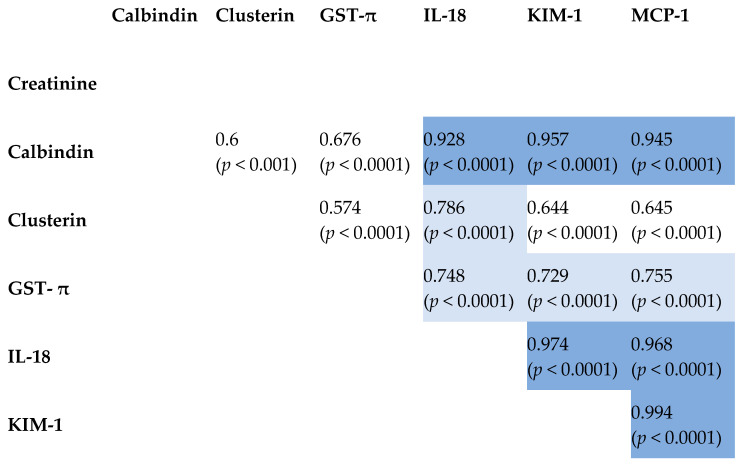
Correlation matrix heatmap. Pearson’s product–moment correlation coefficients along with raw *p*-values calculated for the six parameters in the group of pediatric patients with CKD.

**Figure 3:**
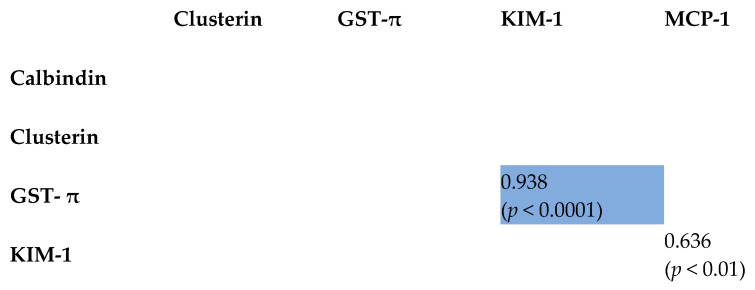
Correlation matrix heatmap. Pearson’s product–moment correlation coefficients along with raw *p*-values calculated for the five parameters in the control group.

Comprehensive results are presented in Figure 1, Figure 2 and Figure 3, with reported coefficients having a *p*-value less than 0.05. The intensity of color in the tables corresponds to the strength of the correlation, providing a visual representation of the interrelationships among the biomarkers.

Although the preceding analysis hinted at the lack of statistical expressibility of creatinine levels in relation to the other parameters, a formal multiple regression was conducted to confirm this observation. The results validated the initial intuition, indicating that creatinine levels cannot be adequately expressed as a multilinear function dependent on calbindin, clusterin, GST-π, KIM-1, and MCP-1.

## 3. Discussion

The deterioration of kidney function among children who have survived Wilms tumor is due to the treatment used in the past, while CKD is a distinct, progressive condition characterized by a gradual loss of kidney function over time. Although WT and CKD are distinct entities, both conditions can significantly impact renal health in pediatric patients. Wilms tumor may lead to renal complications due to its treatment modalities, including nephrectomy and nephrotoxic chemotherapy, which can exacerbate existing renal insufficiency or lead to the development of CKD [16,17]. Biomarkers such as serum creatinine and cystatin C provide valuable insights into glomerular filtration rate (GFR), while urinary markers like neutrophil gelatinase-associated lipocalin (NGAL) and kidney injury molecule-1 (KIM-1) are critical for assessing tubular injury and predicting CKD progression [18,19,20]. In the present study, we sought to elucidate biomarker patterns among pediatric patients with WT and CKD to understand if there are some similarities in the kidney injury ethology.

In our study, the comparative analysis of biomarker levels revealed that creatinine levels were significantly elevated in the CKD group compared to the WT group and the control group. This finding aligns with the existing literature, which consistently identifies elevated serum creatinine as a hallmark of CKD due to its association with decreased GFR and impaired renal function [21]. In contrast, patients with WT typically exhibit less pronounced changes in creatinine levels unless significant renal parenchymal damage or reduced renal mass occurs post-nephrectomy or due to nephrotoxic treatments [22,23].

Although initial analyses of clusterin and GST-π suggested notable differences between CKD and WT patients, these were not statistically significant. This aligns with the current literature highlighting their roles in renal tubular injury and oxidative stress in CKD [24,25]. Clusterin, known for its protective and anti-apoptotic functions, was upregulated in CKD as a response to cellular stress [26].

Upon normalization of urine creatinine, statistically significant differences emerged, underscoring the impact of such normalization in elucidating subtle patterns of renal stress. The elevation in clusterin for CKD was pronounced with a *p*-value of <0.001, affirming its biomarker status in CKD progression. Similarly, GST-π and KIM-1 levels were elevated post-normalization, reinforcing their connection to oxidative stress and kidney injury, respectively. In contrast, Calbindin showed reduced levels in CKD compared to controls, which became significant post-normalization, reflecting its association with renal function integrity. For IL-18, normalization revealed a significant elevation in CKD, aligning with its known pro-inflammatory role in CKD-related immune activation [27,28]. MCP-1 also displayed significant patterns post-normalization, highlighting its chemotactic influence in renal inflammation. The results reinforce previous findings on the necessity of the creatinine normalization to increase biomarkers accuracy and proper interpretation [29,30]. There are several limitations of the study that may explain lack of significance in biomarkers level among the groups. The relatively small sample size limits the generalizability of the findings and may reduce the statistical power to detect significant differences between the groups. Furthermore, the assessment of biomarkers was restricted to a select number of indicators, potentially overlooking other relevant biomarkers that could provide additional insights into renal function and injury. The cross-sectional design of the study precludes the assessment of longitudinal changes and the establishment of causality. Additionally, the single-center nature of the study may introduce site-specific biases, and variations in patient demographics or clinical practices could influence the results. Importantly, the study did not collect detailed etiological data at baseline, which limits the understanding of the cohort’s heterogeneity, and the generalizability of the findings related to the underlying causes of CKD in the enrolled patients.

The strengths of this study include the use of age- and sex-matched cohorts, which enhance the comparability of the groups and reduce confounding variables. Additionally, all samples were collected at the same clinic and analyzed in the same laboratory, thereby minimizing experimental bias and ensuring consistency in the data collection and analysis processes.

This study examined the positive correlations between various biomarkers in WT, highlighting their implications for kidney injury. GST-π and calbindin showed a positive correlation, which might suggest a coordinated increase in response to cellular stress and damage in the kidney [31,32]. Calbindin and KIM-1 were also positively correlated, indicating that calcium signaling disruptions may lead to renal tubular injury, thereby upregulating KIM-1 [20,33]. The correlation between calbindin and MCP-1 may suggest that calcium signaling alterations are linked with inflammatory processes in Wilms tumor [34,35]. Similarly, the positive correlation between clusterin and KIM-1 indicated a concerted response to kidney injury, with both proteins working to mitigate damage and promote repair [36,37]. GST-π’s positive correlation with KIM-1 and MCP-1 highlighted the interconnected processes of oxidative stress, tubular injury, and inflammation in WT [18,20,38,39]. These relationships underscore the complex interplay between oxidative stress, calcium signaling, inflammation, and tissue injury in the kidney, providing insights into the mechanisms underlying kidney function.

This study also explored positive correlations among various biomarkers in pediatric CKD, providing insights into kidney injury mechanisms. The positive correlation between calbindin and IL-18 might suggest that disruptions in calcium signaling could be linked to inflammatory responses, given that IL-18 serves as a pro-inflammatory cytokine [31]. Calbindin and KIM-1 were also positively correlated, indicating that calcium signaling disruptions may lead to renal tubular injury, thereby upregulating KIM-1 [20]. The correlation between calbindin and MCP-1 might indicate that changes in calcium signaling are associated with inflammatory processes in pediatric CKD [34].

Clusterin and IL-18 were positively correlated, indicating a relationship between cellular stress responses and inflammation, given clusterin’s role in tissue remodeling and apoptosis [36]. Similarly, the positive correlation between clusterin and KIM-1 might imply a coordinated response to kidney injury, where both proteins possibly function to mitigate damage and promote repair [37]. The positive correlation between clusterin and MCP-1 highlighted the interconnected processes of cellular stress and inflammation [35].

GST-π and IL-18 showed a positive correlation, linking oxidative stress to inflammatory responses in pediatric CKD [38]. GST-π’s positive correlation with KIM-1 highlighted the interconnected processes of oxidative stress and tubular injury [39]. Additionally, GST-π and MCP-1 showed a positive correlation, which might suggest that oxidative stress could be driving inflammation in pediatric CKD [32].

IL-18 and KIM-1 were positively correlated, indicating that inflammatory responses and tubular injury are interconnected processes in pediatric CKD [33]. The positive correlation between IL-18 and MCP-1 might suggest that inflammation plays a significant role in kidney injury [40]. Finally, KIM-1 and MCP-1 were positively correlated, underscoring the relationship between tubular injury and inflammation in pediatric CKD [41].

This study highlighted positive correlations between some biomarkers in both WT and pediatric CKD, indicating the possibility of shared mechanisms of kidney injury. In both conditions, the positive correlation between calbindin and KIM-1 might suggest that disruptions in calcium signaling could lead to renal tubular injury and the upregulation of KIM-1 [20,33]. Calbindin’s correlation with MCP-1 in WT and pediatric CKD indicates that calcium signaling alterations are linked to inflammatory processes [34,35]. Clusterin and KIM-1 exhibited similar positive correlations in both diseases, which might suggest a coordinated response to mitigate damage and promote repair [36,37]. GST-π’s positive correlation with KIM-1 and MCP-1 in both conditions highlights the interconnected processes of oxidative stress and tubular injury [18,20,38,39].

Our data provide insights into the shared kidney pathology of CKD and WT, underscoring the need for deeper investigation. Considering the cross-sectional nature of our study, we suggest future longitudinal cohort studies that track patients over time. These studies could validate our biomarker associations, reveal dynamic changes, and clarify causal pathways, ultimately informing therapeutic strategies and improving patient care.

## 4. Materials and Methods

Study Population and Sample Collection: Twenty pediatric survivors of WT after unilateral nephrectomy (stages I–IV), forty pediatric patients with CKD due to nephrological diseases, and twenty healthy volunteers were enrolled in this study. Participants aged between 1 and 18 years were included. The matching of cohorts by sex and age was moderate, allowing for a balanced comparison while acknowledging the natural variations within the sample population. To minimize confounding factors related to diurnal variations and hydration status, urine samples were collected under standardized conditions. All samples were obtained as first-morning void specimens. Participants were instructed to avoid excessive fluid intake the evening before and morning of sample collection. They were also asked to refrain from strenuous physical activity for 24 h prior to sample collection. All samples were collected at the time of clinical stage assessment. Specifically, samples from WT patients were obtained during a state of stable remission, while CKD samples were collected as part of routine evaluation at the respective clinical stage.

Biomarker Analysis: The commercially available Bio-Plex Pro RBM Kidney Toxicity Assay (catalog number 171ATR1CK, Bio-Rad Laboratories, Hercules, CA, USA) is built on magnetic beads that enable simultaneous quantification of six proteins in each well of a 96-well plate. The Bio-Plex assay was performed on human urine samples according to the manufacturer’s instructions. The assay principle was based on the reaction with an antibody against a specific protein which was covalently coupled to fluorescently dyed magnetic beads, each with a distinct wavelength specific to the target protein. The bead-conjugated antibodies reacted with the sample containing the protein of interest. After the series of washes, to remove unbound protein, biotinylated antibodies optional for different epitopes of the target proteins were added to the reaction. The final complex was made by adding a streptavidin–phycoerythrin (SA-PE) conjugate. A Bio-Plex 200 Reader (Bio-Rad Laboratories, Hercules, CA, USA) which is a dual-laser, flow-based microplate reader, detected the internal fluorescence of the individually dyed beads and the intensity of the signal on the bead surface. The obtained signal was expressed as median fluorescence intensity (MFI), analyzed, and presented as concentration (pg/mL) by the Bio-Plex Manager Software (Bio-Rad Laboratories, Hercules, CA, USA). The concentration of the analyte attached to the individual beads was proportional to the MFI of the phycoerythrin signal.

Clinical Data Collection: Alongside biomarker assessments, comprehensive clinical parameters were recorded for all participants. These encompassed blood and serum creatinine levels, body mass index (BMI), anthropometric measurements (height and weight), serum albumin levels, vitamin D3 levels, parathyroid hormone (PTH) levels, calcium levels, phosphorus levels, and alkaline phosphatase (ALP) levels. The estimated glomerular filtration rate (eGFR) was calculated using the updated Schwartz formula as eGFR (mL/min/1.73 m^2^) = 0.413 × (height in cm/sCr in mg/dL).

Statistical Analysis: Statistical analyses were conducted to compare biomarker levels and clinical parameters across the WT, CKD, and healthy control groups. Descriptive statistics were employed for data summarization, while inferential statistics, including *t*-tests or ANOVA, were utilized for group means comparisons. Correlation analyses were also performed to investigate associations between biomarkers and clinical parameters between the three analyzed groups.

## Figures and Tables

**Table 1:** Clinical characteristics of the study groups.

	WT	CKD	Healthy Control
Patients (n, %)			
- Male	7 (35%)	18 (45%)	10 (50%)
- Female	13 (65%)	22 (55%)	10 (50%)
Median age (years, range)	13 (4–18)	9 (1–16)	NA
Disease advancement:			
Stage of Wilms Tumor at diagnosis			
- Total	20		
- stage I	7 (35%)		
- stage II	4 (20%)		
- stage III	5 (25%)		
- stage VI	4 (20%)		
Stage of CKD among WT survivors (n, %)			
- stage I	4 (20%)		
- stage II	16 (80%)		
Stage of Chronic Kidney Diseases among children with nephrological diseases (n, %)			
- Total	40		
- stage I	14 (35%)		
- stage II	6 (15%)		
- stage III	4 (10%)		
- stage IV	4 (10%)		
- stage V	12 (30%)		

**Table 2:** Means and standard deviations (given in brackets) of the parameters of kidney damage in the three analyzed groups.

	Creatinine (mg/dL)	eGFR (mL/min/1.73 m^2^)	Calbindin (pg/mL)	Clusterin (pg/mL)	GST-π (pg/mL)	IL-18 (pg/mL)	KIM-1 (pg/mL)	MCP-1 (pg/mL)
**WT**	0.64 (0.26)	131.6 (62.16)	59.8 (57.1)	40.8 (70)	0.847 (1.3)	0.012 (0.004)	0.244 (0.314)	0.128 (0.122)
**CKD**	1.32 (1.9)	62.35 (50.55)	39.6 (81.8)	349 (1310)	5.55 (20.6)	0.054 (0.249)	0.378 (1.61)	0.517 (0.288)
**Control**	0.445 (0.11)	139.94 (54.65)	75.2 (87.4)	18.1 (21)	5.24 (10)	0.04 (0.04)	0.173 (0.168)	0.169 (0.111)
***p*** **-value**	<0.05	<0.05	0.42	0.38	0.56	NA	0.8	0.52

**Table 3:** Normalization of urine biomarkers to creatinine levels across experimental groups.

	Calbindin/cr.	Clusterin/cr.	GST-π/cr.	IL-18/cr.	KIM-1/cr.	MCP-1/cr.
**WT**	0.237 (0.129; 0.808)	0.114 (0.013; 0.872)	0.007 (0.002; 0.013)	0.0001 (0.00009; 0.0003)	0.001 (0.0005; 0.0047)	0.0009 (0.0007; 0.002)
**CKD**	0.348 (0.086; 0.601)	0.475 (0.109; 6.607)	0.004 (0.002; 0.015)	0.0002 (0.0001; 0.0004)	0.003 (0.001; 0.004)	0.003 (0.001; 0.013)
***p***	<0.001	<0.001	<0.001	0.009	<0.001	<0.001

Data are given as median and interquartile range (IQR).

## Data Availability

The data that support the findings of this study are not openly available due to reasons of sensitivity and are available from the corresponding author upon reasonable request. Data are located in controlled access data storage at the institutions participating in the study.
